# Dr. Bartlett’s Case of Typhoid Fever in a Patient Sixty-Three Years Old

**Published:** 1842-09-24

**Authors:** Elisha Bartlett


### ANALECTA.

*Typhoid Fever in a Patient sixty-three years old.* By Elisha Bartlett, M. D.—On Wednesday, the 24th of August, I was invited by Dr. Butterfield, of this city, to attend the examination of a female subject, sixty-three years old, who had died twenty-four hours previously under the care of Dr. Huntington. Dr. Huntington was unable to be present. Dr. Butterfield had seen but little of the patient; and from the very partial history, which we had then had of the symptoms, we supposed it most probable that the case had been one of disease of the brain. As the case is one of considerable interest, I shall report it in the order which, under the circumstances, we were obliged to pursue, in its investigation and diagnosis.

The organ first examined was the brain. We found, here, moderate venous congestion, and pretty abundant sub-arachnoid serous effusion. The ventricles, also, contained from half an ounce to an ounce each of clear, colourless serum. The colour and consistence of the brain were in no way obviously changed.

The adipose tissue upon the abdomen was from an inch and a half to two inches in thickness; and the omentum was very much loaded with fat. The large intestine was fully distended with flatus, and the small intestines moderately so. The kidneys were somewhat softened, perhaps, but in no way very obviously altered from their natural condition. As soon as the small intestines were exposed, I was struck with the very marked, dark-coloured appearance of several of Peyer's glands, and with their very manifest thickening; and I remarked to Dr. Butterfield, that, unexpected as the discovery might be to us, we were likely, I thought, to find the lesion of typhoid fever. A portion of the lower part of the ileum, about seven feet in length, was removed from the body, slit open, washed, carefully examined, and notes of its condition taken at the time. Eighteen inches from the upper end of the removed portion we found a small oval patch very considerably thickened, with prominent edges, and its surface roughened and abraded. At the distance of two inches from this we found another and larger gland, more thickened than the first, and of a mottled red color. Then, at intervals of from two to fifteen inches, until arriving at within a foot of the ileo-cæcal valve, we found eight eliptical plates, several of them quite large, one of them two and a half inches long by one inch wide; more or less extensively diseased. Most of them were of a dark-red colour; they were much thickened, with prominent edges; some of them were extensively and deeply ulcerated, besides being the seat of the peculiar yellow deposition or degeneration so common in this lesion. The last ten or twelve inches of the intestine were thickly scattered over with smaller and somewhat more irregularly-shaped patches, in a condition similar to those which have just been described. This state of things terminated abruptly at the ileo-cæcal valve. Several of the isolated follicles of the ileum were red and thickened. The intervening mucous membrane was but little altered from its natural state. There were a few isolated follicles of the cœcum slightly prominent and reddened. The contents of the intestines were yellow and pultaceous. Several of the mesenteric glands, corresponding to the lower extremity of the ileum, were much enlarged, one of them to the size of the end of the thumb, softened, and of a reddish-yellow colour. The spleen was enlarged, dark and softened. The consistence of the liver, also, was very decidedly diminished. The upper portion of the alimentary tube was not examined.

The heart was softened, somewhat, though otherwise natural. There were no pleuritic adhesions, and the entire lungs were perfectly healthy, excepting that the lower and posterior edge of the right lung had undergone the kind of *carnification* common in typhoid fever. It was of a dark, bluish-red colour; wholly destitute of air; not granular or hepatized; with a tough flabby texture, not easily torn or penetrated by the fingers.

Here, then, was a case, so far as the evidence to be derived from the morbid appearances of the organs could settle the question, of typhoid fever; not doubtfully or vaguely, but, on the contrary, very clearly and unequivocally marked. It remained to be seen, whether, contrary to what we had been led to suppose, this diagnosis, founded exclusively upon the autopsy, would be justified by the history of the case during life. On inquiry of the friends of the patient, and of her physician (Dr. Huntington,) it was found that she was attacked on Monday, the 15th of August, after breakfast, with severe pain in the head; and that she complained of being very cold. She ate no dinner, but kept about during the day. Through the night she was dozy, but restless; and during the whole of the next day, she remained in bed, with increasing headache, and with pains over the whole body, but especially severe in the back and hips. She had no appetite, was thirsty, and inclined to sleep, though restless. This state of things continued through Tuesday night, with the addition of slight wandering delirium. On Thursday evening there was much increase of the somnolence, so that for some hours she could not be roused. From this period up to the fatal termination of the case, there was drowsiness, dizziness, restlessness, constant pulling at the bed-clothes, delirum; and marked rigidity of the muscles of both arms, constituting, as is now well known, an almost invariably fatal symptom in this disease. For the last few days there were four or five liquid discharges from the bowels daily, of a dark yellowish colour. The pulse was rapid, and during the last four or five days, irregular and intermittent. The tongue was nearly natural throughout the disease. Death took place about noon on Tuesday, the 23d of August, eight days from the commencement of the fever.

This patient had been residing, since the first of March, about seven miles from her usual home, in a neighborhood where typhoid fever had been somewhat prevalent during the summer. One of her own grandchildren was sick with the disease from the 8th to the 21st of June, in the house where she lived.

The principal interest of this case is connected with the age of the patient. It is now well settled that typhoid fever is, in a great majority of instances, a disease of early life. Of one hundred and thirty-eight cases, the histories of which are analyzed in Louis's Researches, only ten were over thirty years old, and no one was over forty. Of one hundred and forty-seven cases cited by Chomel, only six were over forty years old, and only one was over fifty. The last-mentioned observer says, in his Clinical Lectures on Typhoid Fever, published in 1834, that, so far as was then known, no case had been seen in which the patient was over fifty-five years of age. The alleged instances, reported by Andral, are none of them authentic cases of the disease. Of two hundred and ninety-one cases analysed in Dr. Jackson's report, only sixteen were thirty-five years old or upwards. The extreme age is not mentioned. In a memoir by Gendron upon this disease in one of the French provinces, three cases are mentioned in which the patients were between fifty and sixty years old; and four in which they were between sixty and seventy-five years of age. I have ventured to make the suggestion, in a work upon this disease and upon typhus fever, now in the press, that typhoid fever may be found to occur more frequently after the fortieth year of life in the country, than in large towns and cities. This question can be settled only by the careful observations of the future; and be it as it may, I have thought it worth while to place on record the present *authentic and unequivocal example of typhoid fever, or dothinenteritis, occurring in the country, in a patient passed the sixty-third year of life.—Boston Med. and Surg. Journ.*, Sept. 7, 1842.

[We agree with Dr. Bartlett that persons advanced in life are more apt to be attacked with typhoid fever in the country than in town. The only patient, more than forty years of age, whom we remember seeing, was a gentleman fifty-four years of age, who was taken with the fever in the month of March, in a local epidemic which prevailed a few miles west of this city. In that epidemic, by far the larger number of patients were young people.

The fact of the disease prevailing as an epidemic is one cause, however, why it extends itself to persons advanced in life. In the city of Boston, in the epidemic of 1833, we were informed by an eminent physician that persons much advanced in life were taken with the fever. We do not, however, remember to have heard of a case in which the age of the patient was as great as in the instance reported by Dr. Bartlett. In typhus it is well known that the contrary rule holds good.—W. W. G.]

